# Prognostic Role of S100A8 in Human Solid Cancers: A Systematic Review and Validation

**DOI:** 10.3389/fonc.2020.564248

**Published:** 2020-11-09

**Authors:** An Huang, Wei Fan, Jiacui Liu, Ben Huang, Qingyuan Cheng, Ping Wang, Yiping Duan, Tiantian Ma, Liangyue Chen, Yanping Wang, Mingxia Yu

**Affiliations:** ^1^ Department of Clinical Laboratory, Zhongnan Hospital of Wuhan University, Wuhan, China; ^2^ Key Laboratory of Carcinogenesis andTranslational Research (Ministry of Education), Department of Gastrointestinal Surgery III, Peking University Cancer Hospital & Institute, Beijing, China; ^3^ Department of Obstetrics and Gynecology, Hubei Provincial Hospital of TCM, Wuhan, China

**Keywords:** S100A8, cancer, prognosis, meta-analysis, validation

## Abstract

**Background:**

S100A8 plays a key role in many cellular processes and is highly expressed in various solid cancers. However, the prognostic role of S100A8 has not been well defined. Therefore, we conducted a quantitative meta-analysis to investigate whether or not S100A8 could be used as a prognostic biomarker in solid tumors.

**Methods:**

PubMed, Web of Science, Embase, and Cochrane library were searched to acquire relevant studies that evaluated the association between expression of S100A8 and prognosis of cancer patients. Pooled hazard ratios (HRs) with their corresponding 95% confidence intervals (CIs) were extracted to evaluate the association between S100A8 overexpression and Overall Survival (OS), Disease-Free Survival (DFS), Recurrence-Free Survival (RFS), and Progression-Free Survival (PFS). The expression of S100A8 was also validated by Flow cytometry, immunohistochemistry (IHC), and western blot.

**Results:**

A total of 2,817 patients from 13 independent studies, ranging from 43 to 1,117 patients in size, were statistically analyzed. Our results indicated that a high level of S100A8 expression was significantly associated with poor OS, poor DFS, and poor PFS/RFS. In term of clinical pathological characteristics, a high expression level of S100A8 was significantly associated with differentiation grades, lymphatic metastasis, ER statue, and PR statue. The validation studies showed that the expression of S100A8 was at high levels in MDA-MB-231 (79.7%), MDA-MB-453 (89.2%), HTB-9 (70.2%), and T24 (53.3%) cells and it was higher in breast cancer tissue and bladder cancer tissue than their corresponding para-carcinoma tissue.

**Conclusions:**

S100A8 overexpression was significantly associated with poor clinical prognosis in cancer patients. S100A8 is potential a prognostic biomarker in breast cancer and bladder cancer. More well-designed studies with adequate prognostic data are needed to confirm the prognostic role of S100A8 revealed in this study.

## Introduction

Cancer, the second cause of death globally, is one of the leading causes of morbidity and mortality worldwide (1). According to the International Agency for Research on Cancer (IARC), there are approximately 18.1 million new cancer cases and 9.6 million cancer deaths worldwide in 2018 (2). Despite the remarkable progress in various treatment strategies for cancer, such as surgery, radiotherapy, chemotherapy, immunotherapy, and targeted therapy, the prognosis of many cancer patients is still unsatisfactory, mainly owing to the local recurrence and distant metastasis (3, 4). At present, the prognosis of cancer patient is made mainly based on the TNM staging of AJCC and UICC. However, patients in the same stage may still have greatly different prognosis. To develop the most effective individualized treatment strategy for cancer patients and improve clinical outcomes, reliable prognostic biomarkers are extremely useful.

With the first member discovered in 1965, S100 family members are small, acidic-Ca2+ binding proteins that are involved in a wide range of biological processes (5). Most of the S100 proteins undergo a conformational change to bind to Ca2+ and regulate the homeostatic Ca2+ homeostasis, cell cycle, cell growth and migration, cell scaffold composition, and transcriptional molecular regulation. The S100A8 is a calcium-binding site of the EF-hand type with a low calcium-binding affinity site at the N terminus (N-terminal EF-hand; EF-hand I) and a high affinity site at the C terminus (C-terminal EF-hand; EF-hand II) (6, 7). S100A8 plays an important role in the regulation of immune response and inflammatory processes. It is mainly expressed in bone marrow-derived immune cells, such as macrophages and neutrophils (5, 8, 9). S100A8 acts in a cytokine-like behavior, by binding to cell surface receptors that trigger signaling pathways to take part in the inflammatory process, and plays a key role in many cellular processes, including cell survival, cell cycle progression, differentiation, proliferation, and cell migration (10, 11).

S100A8 has been found to be highly expressed in a variety of inflammation-related diseases, such as inflammatory bowel disease (12). Recent publications also have indicated that S100A8 is highly expressed in various solid cancers, including breast cancer (13–19), oral squamous cell carcinoma (20), prostate cancer (21, 22), bladder cancer (23–29), gastric cancer (30–32), lung cancer (33, 34), and liver cancer (35, 36). Most of the studies suggested that an overexpression of S100A8 was correlated with low survival rate in cancer patients. However, single study may be not accurate and convincing. Thus, it is helpful to probe the role of S100A8 using meta-analysis of a much larger number of patients from the literature reports to better understand the potential clinical prognostic value of S100A8 in solid tumor.

## Methods

Our meta-analysis was accomplished on the basis of the Preferred Reporting Items for Systematic Reviews and Meta-Analyses (PRISMA) statement (37).

### Literature Search Strategy

We searched PubMed, Web of Science, Embase, and Cochrane library to acquire relevant studies with language restriction to English from January 1, 2000 to April 1, 2019, but without restrictions on geographic areas. The following retrieval strategy was used:

(“cancer” OR “tumor” OR “tumour” OR “neoplasm” OR “carcinoma” OR “adenocarcinoma”) AND (“S100A8” OR “Calgranulin A” OR “MRP-8” OR “S100 calcium binding protein A8”) AND (“prognosis” OR “prognostic” OR “outcome”). References in each manuscript were also manually screened to identify more relevant articles.

### Study Inclusion Criteria

Two authors independently scrutinized the hits for articles that met the following criteria: (1) studies explored the relationship between S100A8 and the prognosis of patients with cancers; (2) expression of S100A8 was measured in the tumor tissues; (3) studies presented sufficient data to calculate the survival data, including hazard ratio (HR) with 95% confidence interval (CI) or odds ratio (OR); (4) only the most integrated ones were included for articles with duplicated or overlapping study population;

### Data Extraction

The following data was independently extracted by two authors from each paper: (1) general information including first author, publication year, country, sample size, cancer types, and the follow-up duration; (2) detection methods and cut-off values; (3) HRs and 95% CIs investigating the relationship between the expression of S100A8 and OS or DFS or RFS or PFS; (4) clinicopathological characteristics including gender, TNM stage, differential grade, and lymph node metastasis. Any differences were resolved through discussion. The Engauge Digitizer 4.1 software (38, 39) was used to extract data from the Kaplan-Meier curves in articles that did not directly provide HRs and 95% CIs. Tumor cell differentiation grade was subdivided as poor and well/moderate differentiation. TNM stage was dichotomized as III/IV and I/II. In addition, the quality of the included studies was assessed using the Newcastle Ottawa Scale (NOS) (40), with scores ≥7 considered as high quality article.

### Statistical Analysis

The prognostic significance of the S100A8 expression in OS, DFS, RFS, and PFS were analyzed through combining HR and 95% CI. The association between S100A8 expression and clinic pathological features were assessed by the combination of OR with 95% CI. The χ^2^ based Q test and the I^2^ test were undertaken to assess the statistical heterogeneity in included studies. When combining the data, a fixed-effect model was used if there was no remarkable heterogeneity (I^2^ < 50% or P-value > 0.05). Otherwise, a random-effect model was applied. All statistical tests were two-sided and P < 0.05 was considered statistically significant. Begg’s funnel plot was used to assess publication bias. All statistical analyses were conducted using the STATA software (version 12.0, Stata Corp, College Station, TX, USA).

### Flow Cytometry

Cells were detached with trypsin–EDTA (Invitrogen) and re-suspended in PBS containing 1% BSA and 0.1% sodium azide. The samples were incubated for 1 h on ice with anti-S100A8 mAb or the corresponding isotype antibodies as negative controls. After washing with PBS, cell was stained by incubating with fluorescein isothiocyanate (FITC) labeled anti-rabbit immunoglobulin G (IgG; Jackson ImmunoResearch Laboratories) for 1 h. Stained cells were analyzed on a FACSCalibur 440E (BectonDickinson) using Cell Quest software (BD Biosciences Immunocytometry Systems).

### Immunohistochemistry

Breast cancer and bladder cancer tissues were collected from patients in Zhongnan Hospital, Wuhan University. Sections of 4-μm thick, formalin-fixed, and paraffin-embedded tissues from the patients were deparaffinized in xylene and rehydrated in graded ethanol. Following heat-mediated antigen retrieval, S100A8 was detected in the tissues by the avidin–biotin complex method. The sections were incubated for 1 h with the corresponding specific primary antibody (Santa Cruz Biotechnology), followed by washing, another 1-h incubation with biotin-labeled anti-murine IgG antibody (Boyao Biotechnology), washing again, and then incubation with peroxidase-labeled streptavidin for 20 min. Immunostaining was visualized by color reaction to diaminobenzidine for 5 min. As a negative control, isotype antibody was substituted for the primary antibody. Then, the percentages of brown-stained cells indicating the presence of S100A8 was determined. The positive cell ratio (integral optical density value/integral area) was calculated by Tongji Qianping Image Analysis Software.

### Western Blot

Total protein was extracted from frozen tumor tissues in ice-cold lysis buffer (50 mmol/L Tris–HCl pH 7.5, 150 mmol/L NaCl, 5 mmol/L EDTA, 1% Nonidet P-40), containing a protease inhibitor cocktail (Calbiochem) on ice for 15 min. Twenty microgram protein was fractionated by 12% SDS-PAGE and then transferred from the gel onto a polyvinylidene difluoride (PVDF) membrane. After blocking with 5% nonfat milk in PBS–Tween-20 (0.05%) overnight at 4°C, the membrane was then probed with antibodies specific to S100A8 or β-actin (Santa Cruz Biotechnology), followed by a HRP-conjugated secondary antibody against mouse IgG. The enhanced chemiluminescence (ECL) from NEN LIFE Science was used to visualize the antibody reaction. Bands were quantified by a calibrated imaging densitometer (GS-710; Bio-Rad) and analyzed by “Quantity One” software (Bio-Rad).

## Results

### Literature Search

**Figure 1:**
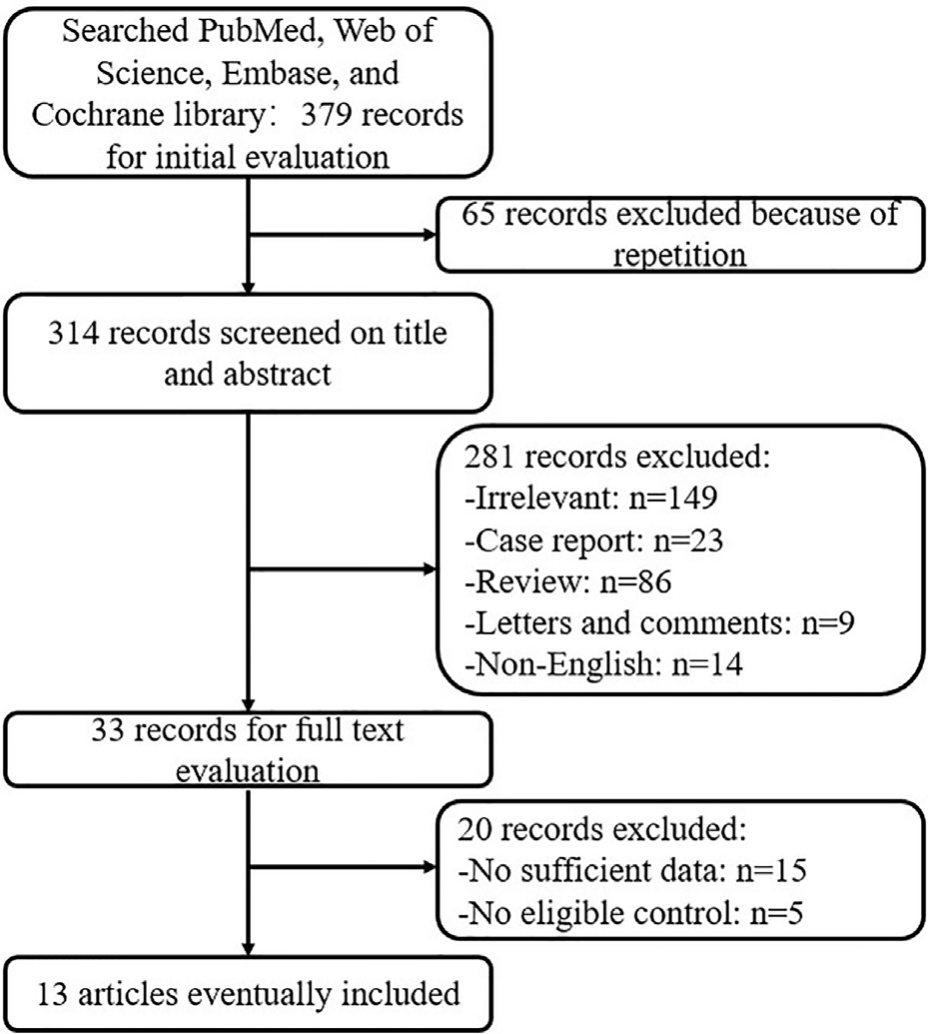
Flowchart of the selection process in the meta-analysis.

Initially a total of 379 articles were identified based on the search strategy. However, only 33 studies met the inclusion criteria. Subsequently, 20 studies were excluded due to either a lack of an appropriate control or insufficient amount of data. Therefore, a total of 13 studies were enrolled in our meta-analysis (13–18, 20, 21, 23, 26, 27, 30, 33). The flowchart of the selection process is summarized in **Figure 1**.

### Study Characteristics

Our meta-analysis included a total of 2,817 patients from 13 studies, with a maximum sample size of 1,117 patients and a minimum sample size of 43. A total of six cancer types were included: breast cancer, oral squamous cell carcinoma (OSCC), prostate cancer, bladder cancer, non-small cell carcinoma of the lung (NSCCL), and gastric cancer. Two studies used RT-PCR and one study used automated quantitative immunoﬂuorescence (AQUA) to evaluate the expression of S100A8, while all other studies assessed the expression of S100A8 by immunohistochemistry (IHC). Among these studies, six studies were on OS, six studies on DFS, four studies on RFS, and two studies on PFS. The detailed characteristics of these eligible studies are listed in **Table 1**.

**Table 1:** Summary of all included eligible studies.

First author	Year	Country	No. of patients	Tumor type	Method	Cut-off	Outcome	Analysis	NOS
Zhang (18)	2017	China	1,117	Breast cancer	IHC	NR	OS	Kaplan-Meier curves	7
Wang (17)	2018	China	140	Breast cancer	IHC	NR	OS/DFS	Multivariate	7
Parris	2013	Sweden	63	Breast cancer	IHC	IHC ≥1+	DFS	Kaplan-Meier curves	8
Parris (16, 20)	2014	Sweden	43	OSCC	IHC	IHC ≥1+	OS	Multivariate	7
Mukhtar (15)	2012	America	116	Breast cancer	IHC	NR	RFS	Kaplan-Meier curves	8
McKiernan (13)	2011	Ireland	295	Breast cancer	RT-PCR	NR	OS/DFS	Univariate	8
Yun	2014	Korea	69	Prostate cancer	IHC	IHC ≥1+	RFS	Multivariate	8
Ha (23)	2010	Korea	103	NMIBC	RT-PCR	171.2 × 10^3^ copies/μl	PFS	Multivariate	9
Minami (26)	2010	Japan	77	Bladder cancer	IHC	IHC score ≥2	RFS	Multivariate	8
Miller (14)	2017	America	417	Breast cancer	AQUA	AQUA score >95.27	OS/DFS	Multivariate	8
Nicklas (27)	2018	Austria	158	NMIBC	IHC	IHC ≥1+	RFS/PFS	Multivariate	8
Koh	2018	Korea	94	NSCCL	IHC	IHC ≥1+	DFS	Kaplan-Meier curves	8
Fan (30)	2012	China	125	Gastric cancer	IHC	Positive cells ≥64	OS	Kaplan-Meier curves	8

IHC, Immunohistochemistry; AQUA, automated quantitative immunoﬂuorescence; RT-PCR, reverse transcriptase polymerase chain reaction; OS, Overall survival; DFS, Disease-free survival; PFS, Progression-free survival; RFS, Recurrent-free survival; NR, Not reported; NOS, Newcastle-Ottawa Scale; OSCC, oral squamous cell carcinoma; NMIBC, non-muscle invasive bladder cancer; NSCCL, non-small cell carcinoma of the lung.

### Increased S100A8 Expression and OS

**Figure 2:**
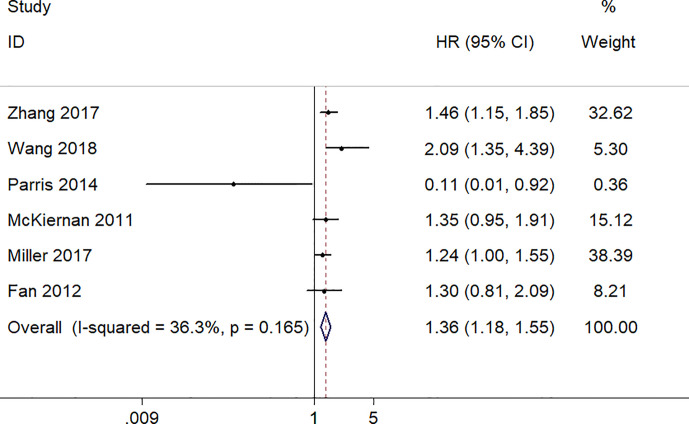
Forest plot of studies evaluating hazard ratios of S100A8 overexpression and the overall survival of cancer patients.

A total of 2,137 patients from six studies with the OS reported were analyzed using the fixed-effect model to estimate the pooled hazard ratio (HR) and corresponding 95% confidence interval (CI) because no obvious heterogeneity was found (I^2^ = 36.3%, p = 0.165). The pooled HR (the high S100A8 expression group versus the low S100A8 expression group) was 1.36 (95% CI 1.18–1.55, p = 0.000). Thus, the result demonstrated that S100A8 overexpression was significantly associated with poor OS in patients with cancers (**Figure 2**).

### Increased S100A8 Expression and DFS

**Figure 3:**
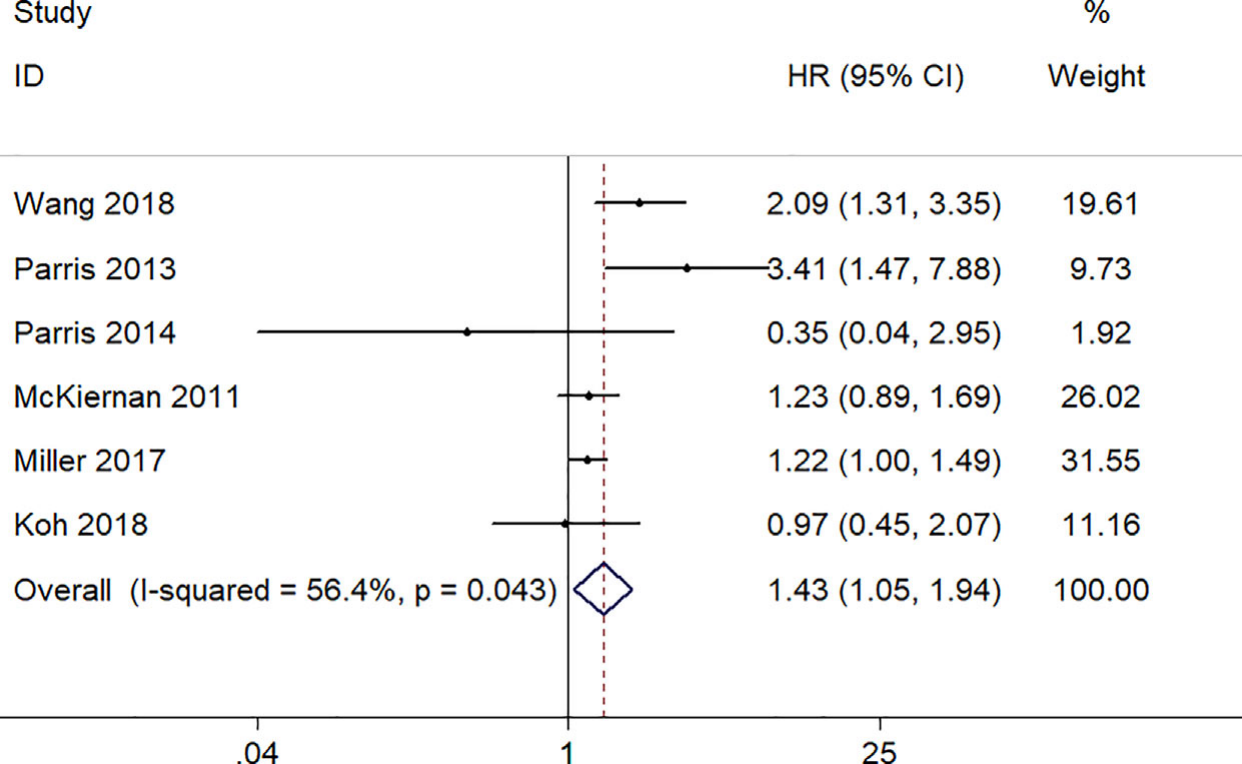
Forest plot of studies evaluating hazard ratios of S100A8 overexpression and the disease-free survival of cancer patients.

There were six studies, involving a total of 1,052 patients, provided appropriate data for DFS analysis. Due to the statistical significance of heterogeneity among these studies (I^2^ = 56.4%, p = 0.043), the random‐effect model was adopted to estimate the pooled HR and corresponding 95% CI. The result indicated that there was a significantly association between high expression level of S100A8 and poor DFS (pooled HR = 1.43, 95% CI 1.05–1.94, p = 0.022) (**Figure 3**).

### Increased S100A8 Expression and PFS/RFS

**Figure 4:**
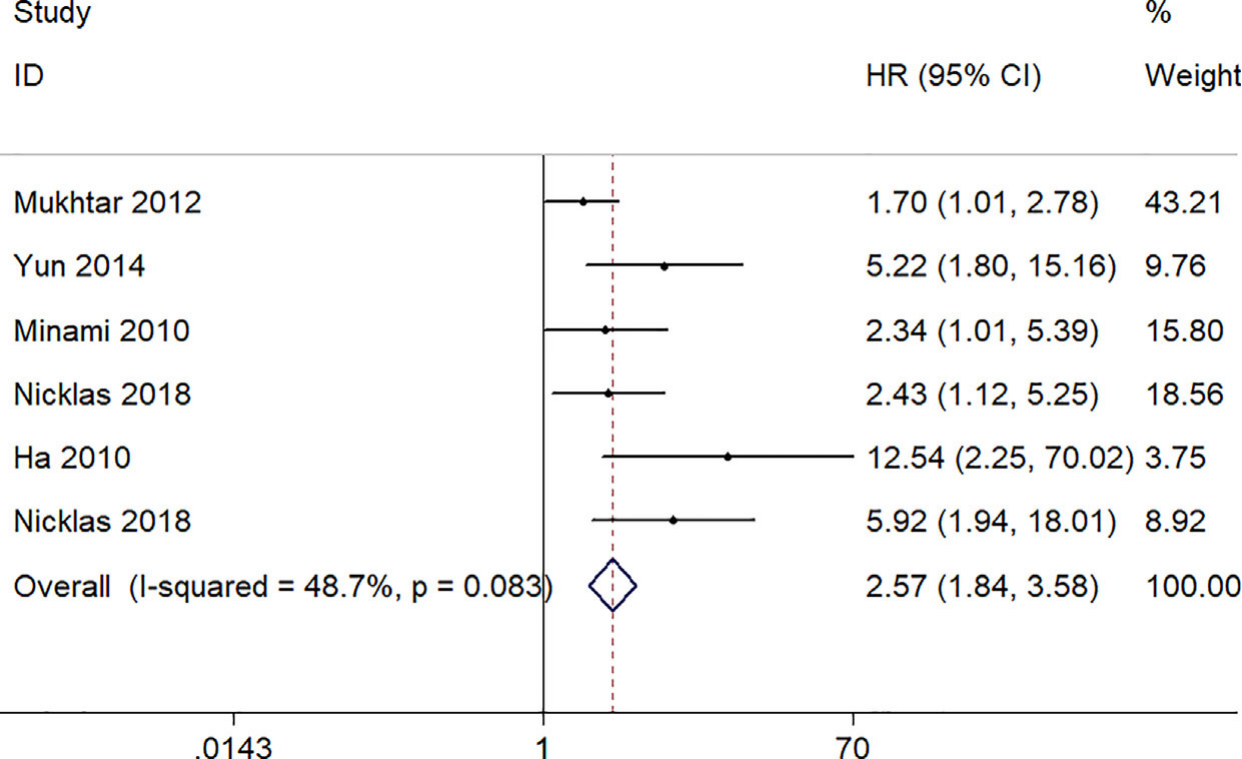
Forest plot of studies evaluating hazard ratios of S100A8 overexpression and the progression-free survival or recurrent-free survival of cancer patients.

A total six studies, involving 523 patients, provided appropriate data for PFS/RFS analysis. The fixed-effect model was used because no obvious heterogeneity was found (I^2^ = 48.7%, p = 0.083). The pooled HR was 2.57 (95% CI 1.84–3.58, p = 0.000), which indicated a significantly association between a high expression level of S100A8 and poor PFS/RFS (**Figure 4**).

### Subgroup Analysis

In order to find possible sources of heterogeneity, we constructed the subgroup analysis. The patients were classified according to their different conditions (**Table 2**). In term of the cancer type, high expression level of S100A8 was significantly associated with poor outcome in breast cancer (pooled HR = 1.43, 95% CI 1.25–1.63) with no significant heterogeneity (I^2^ = 36.8%, p = 0.161) and bladder cancer (pooled HR = 3.90, 95% CI 2.09–7.29) with no significant heterogeneity (I^2^ = 46.6%, p = 0.154). We did not find significant association between S100A8 overexpression and prognosis in other cancers including oral squamous cell carcinoma (OSCC), prostate cancer, non-small cell carcinoma of the lung (NSCCL), and gastric cancer (pooled HR = 1.45, 95% CI 0.68–3.11) with significant heterogeneity (I^2^ = 64.4%, p = 0.038). In term of the analytical method, S100A8 overexpression was significantly associated with poor prognosis in multivariate analysis group (pooled HR = 2.44, 95% CI 1.26–4.70) with significant heterogeneity (I^2^ = 78.0%, p = 0.000). The prognostic significance also could be seen in the Kaplan-Meier curves group (pooled HR = 1.49, 95% CI 1.24–1.79) with no significant heterogeneity (I^2^ = 28.0%, p = 0.235). Similarly, S100A8 overexpression was associated with poor prognosis in the subgroup of sample size.

**Table 2:** Subgroup analysis of pooled HR for OS/DFS/RFS/PFS.

Categories	No. of studies	No. of patients	Pooled HR (95% CI)		Heterogeneity	
			Fix/Random	p-value	I² (%)	P-value
**Cancer type**						
1) Breast cancer	6	2,148	1.43 (1.25–1.63)	0.000	36.8	0.161
2) Bladder cancer	3	338	3.90 (2.09–7.29)	0.000	46.6	0.154
3) Others	4	331	1.45 (0.68–3.11)	0.339	64.4	0.038
**Analysis**						
1) Multivariate	7	1,007	2.44 (1.26–4.70)	0.008	78.0	0.000
2) Kaplan-Meier curves	5	1,515	1.49 (1.24–1.79)	0.000	28.0	0.235
**Sample size**						
1) ≥100	8	2,471	1.43 (1.25–1.62)	0.000	41.1	0.104
2) <100	5	346	1.81 (0.77–4.62)	0.175	72.6	0.006

### Associations Between S100A8 Expression and Clinical Pathological Characteristics

Further, we studied the associations between S100A8 expression and clinical pathological characteristics (**Table 3**), including genders, differentiation grades, TNM stages, lymphatic metastasis, estrogen receptor (ER) status, progesterone receptor (PR) status, and human epidermal growth factor receptor-2 (HER2) status. We found that high expression level of S100A8 was significantly associated with differentiation grades (poorly *versus* well/moderately, OR = 3.91, 95% CI 1.48–10.27, p = 0.006), lymphatic metastasis (N1/N2/N3 versus N0, OR = 1.80, 95% CI 1.17–2.77, p = 0.007), ER status (positive *versus* negative, OR = 0.31, 95% CI 0.21–0.47, p = 0.000), and PR status (positive *versus* negative, OR = 0.29, 95% CI 0.13–0.63, p = 0.002). We did not observe significant association between S100A8 overexpression and genders (male *versus* female, OR = 1.93, 95% CI 0.76–4.89, p = 0.164), TNM stages (III–IV *versus* I–II, OR = 1.34, 95% CI 0.51–3.51, p = 0.554), and HER2 status (positive *versus* negative, OR = 1.91, 95% CI 0.62–5.91, p = 0.263).

**Table 3:** Associations between S100A8 expression and clinical pathological characteristics Sensitivity analysis.

Clinicopathological characteristics	No. of studies	No. of patients	Risk of high S100A8 OR (95% CI)	Significant Z	P-value	Heterogeneity I2 (%)	P-value		Model
Gender (male *vs* female)	4	339	1.93 (0.76–4.89)	1.39	0.164	65.0	0.036	Random effect	
Tumor differentiation (poor *vs* moderate/well)	5	439	3.91 (1.48–10.27)	2.76	0.006	73.4	0.005	Random effect	
TNM stage (III–IV *vs* I–II)	4	402	1.34 (0.51–3.51)	0.59	0.554	69.2	0.021	Random effect	
Lymphatic metastasis (N1/N2/N3 *vs* N0)	5	448	1.80 (1.17–2.77)	2.68	0.007	0.0	0.445	Fixed effect	
ER (positive *vs* negative)	3	319	0.31 (0.21–0.47)	5.71	0.000	0.0	0.443	Fixed effect	
PR (positive *vs* negative)	3	319	0.29 (0.13–0.63)	3.14	0.002	58.5	0.090	Random effect	
HER2 (positive *vs* negative)	3	319	1.91 (0.62–5.91)	1.12	0.263	75.8	0.016	Random effect	

### Sensitivity Analysis

**Figure 5:**
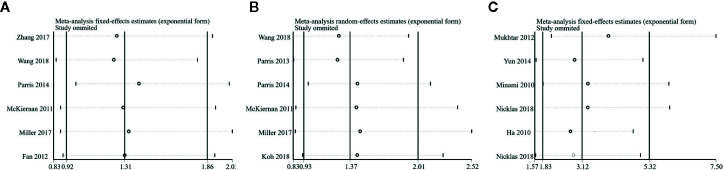
Sensitivity analysis of this meta-analysis. **(A)** Overall survival (OS). **(B)** Disease-Free Survival (DFS). **(C)** Progression-free Survival or Recurrence-Free Survival (PFS/RFS).

To test the reliability of our results, we removed an individual study at a time and estimated the pooled HRs of the remaining studies. No single study dominated our meta-analysis (**Figure 5**) which illustrated that our meta-analysis was stable and credible.

### Publication Bias

**Figure 6:**
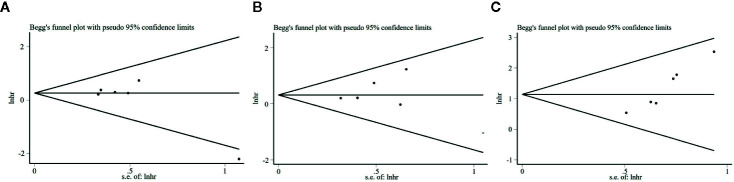
Begg’s funnel plots for the studies involved in the meta-analysis. **(A)** Overall survival (OS). **(B)** Disease-Free Survival (DFS). **(C)** Progression-free Survival or Recurrence-Free Survival (PFS/RFS).

An evaluation of the publication bias by the Begg’s funnel plots revealed no obvious asymmetry in the funnel plot that evaluates the relationship between S100A8 expression and OS (**Figure 6A**). The P value of Egger’s test (P = 0.104) also indicated no obvious publication bias. The result was the same with DFS (P = 0.827, **Figure 6B**). However, publication bias was found in association between S100A8 expression and PFS/RFS (P = 0.002, **Figure 6C**).

### Validation

#### Flow Cytometry

**Figure 7:**
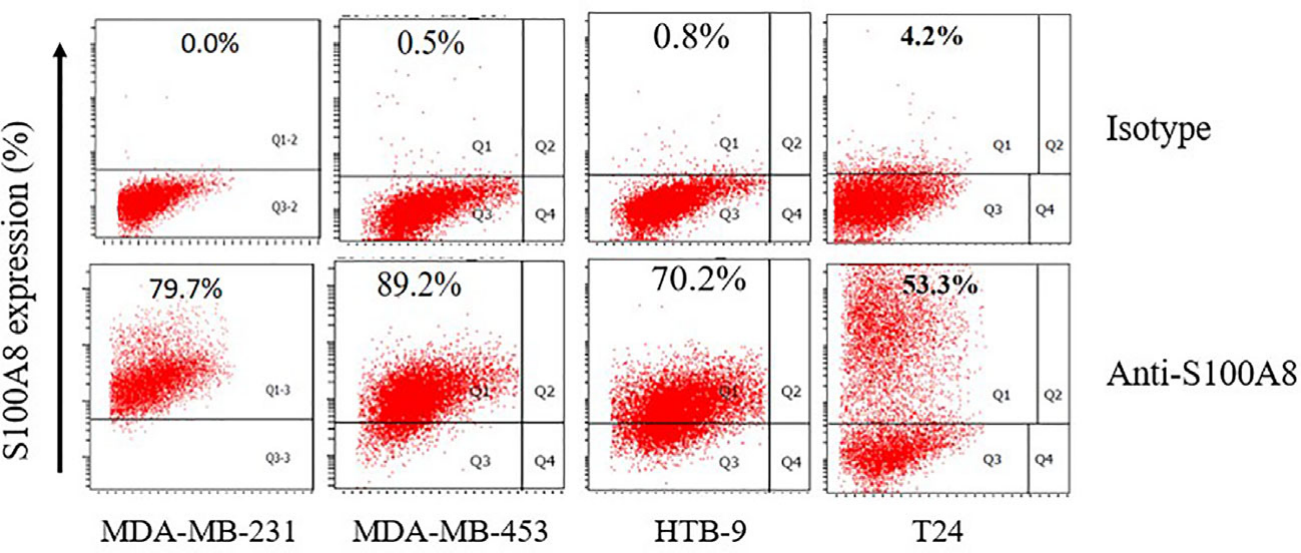
The expression of S100A8 in two human breast cancer cell lines and two human bladder cancer cell lines.

We analyzed the expression of S100A8 in two human breast cancer cell lines (MDA-MB-231 and MDA-MB-453) and two human bladder cancer cell lines (HTB-9 and T24). As is shown, S100A8 expressions were at high levels in MDA-MB-231, MDA-MB-453, HTB-9, and T24 cells (79.7, 89.2, 70.2, and 53.3%, respectively) (**Figure 7**).

#### Immunohistochemistry

We collected 112 patients pathologically diagnosed with primary breast cancer between June 2008 and June 2009 at the Department of Pathology, Zhongnan Hospital of Wuhan University (Wuhan, China) (**Table 4**).

**Table 4:** Clinical pathological characteristics of breast cancer patients (n = 112).

Clinical pathological characteristics	Number of cases (%)
Age (years) (median 50, range 35–72)	
<50	56 (50)
≥50	56 (50)
Tumor grade	
I–II	75 (67.0)
III	37 (33.0)
TNM stages	
I–II	66 (58.9)
III–IV	46 (41.1)
Lymph node metastasis	
Yes	60 (53.6)
No	52 (46.4)
Tumor size	
<5 cm	77 (68.8)
≥5 cm	35 (31.2)

**Figure 8:**
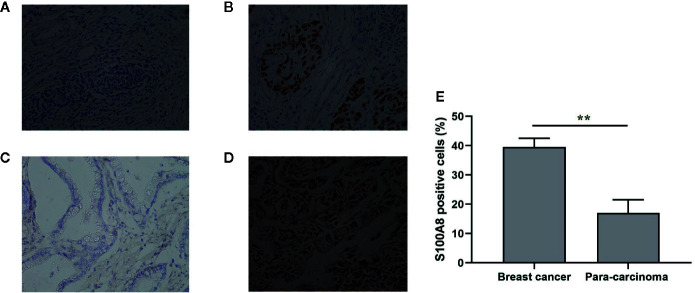
Immunohistochemical examination of the expression of S100A8. **(A)** Para-carcinoma tissue of breast cancer (female, aged 51, magniﬁcation, ×200); **(B)** breast cancer tissue (female, aged 51, magniﬁcation, ×200); **(C)** para-carcinoma tissue of bladder cancer (male, aged 69, magniﬁcation, ×200); **(D)** bladder cancer tissue (male, aged 69, magniﬁcation, ×200). **(E)** The proportion of S100A8 positive cells in breast cancer and its corresponding para-carcinoma tissues (n = 112). **means p < 0.01.

We evaluated S100A8 expression in breast cancer tissues and bladder cancer tissues and their corresponding para-carcinoma tissues using immunohistochemical examination. Tissues were collected from patients in Zhongnan Hospital, Wuhan University. We found that S100A8 staining was positive in breast cancer and bladder cancer tissues, but negative in their corresponding para-carcinoma tissues (**Figure 8**). And in breast cancer and its corresponding para-carcinoma tissues, the proportion of S100A8 positive cells was 39.5 and 17% respectively (P < 0.01) (**Figure 8**).

#### Western Blot

**Figure 9:**
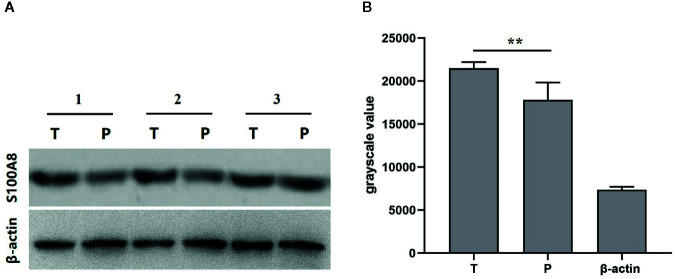
**(A)** Western blot of paired samples of tumor tissue (T) and para-carcinoma tissue (P) immunoblotted against S100A8 or β-actin (used as a loading control). **(B)** grayscale value of western blot of paired samples of tumor tissue (T), para-carcinoma tissue (P), and β-actin (n = 8). **means p < 0.01.

We used random number method to select eight pairs of samples of breast cancer tissues and its corresponding para-carcinoma tissues to analyze the expression of S100A8 using western blot. The grayscale value of western blot of eight paired samples of tumor tissue (T), para-carcinoma tissue (P), and β-actin showed the expression of S100A8 was higher in breast cancer tissues than its corresponding para-carcinoma tissues, which verified that S100A8 could be a predictor of prognosis (**Figure 9**).

## Discussion

Though several studies have been conducted to clarify the role of S100A8 in the occurrence and development of cancers, its potential molecular mechanisms remain unclear. It was shown that S100A8 promoted the invasion of breast cancer cells, depending on advanced glycation end products (RAGE) (41). RAGE binding to S100A8 promoted the phosphorylation of LIN-11, Is11, and MEC-3 protein domain kinase, as well as cofilin. It is a critical step in actin polymerization and cofilin recycling. At the same time, RAGE binding to S100A8 strengthened cell mesenchymal properties and mediated epithelial–mesenchymal transition (EMT). Furthermore, EMT is a process in which epithelial cell layers lose polarity together with cell to cell contacts, leading to a dramatic remodeling of the cytoskeleton. Hence, it plays an significant role in tumor metastasis (42). In terms of mechanism, RAGE bind to S100A8 stably through the NF-κB signaling pathway. S100A8 has binding sites for NF-κB (43), the ligation of cell surface receptors by S100A8 in inflammation can lead to a positive feedback loop and sustained cellular activation, thereby promoting tumor development (41). Another study (44) found that S100A8-induced cell migration and invasion was inhibited by metalloproteinase 2 (MMP2) siRNA and MMP12 siRNA, which indicated that MMP2 and MMP12 were related to the S100A8-induced cell migration and invasion. Additionally, S100A8 caused an increase in MMP2 and MMP12 expression, which could be inhibited by SB203580 (p38 MAPK inhibitor) and Bay (NF-κB inhibitor). As a result, it suggested that S100A8 promoted cell migration and invasion through p38 MAPK-dependent NF-κB activation resulting in an increase of MMP2 and MMP12 in gastric cancer. Consequently, the overexpression of S100A8 in cancers contributes to the proliferation, metastasis, and invasion of tumors. Recent publications have indicated that the overexpression of S100A8 in various solid cancers is highly correlated with low survival rate in cancer patients, suggesting S100A8 as a potential biomarker for cancer prognosis.

Our meta-analysis was the first meta-analysis systematically assessing the prognostic value of S100A8 in patients with cancers. The results revealed that high level of expression of S100A8 in cancers was significantly associated with poor OS, DFS, and PFS/RFS. Our subgroup analysis demonstrated that high expression level of S100A8 was significantly associated with poor outcome in breast cancer and bladder cancer. Further, our results showed that high expression level of S100A8 was significantly associated with differentiation grades, lymphatic metastasis, ER, and PR. We did not observe significant association between S100A8 overexpression and genders, TNM stages, and HER2. The validation data showed that the expression of S100A8 was at high levels in MDA-MB-231, MDA-MB-453, HTB-9, and T24 cells (79.7, 89.2, 70.2, and 53.3%, respectively) and was higher in breast cancer tissue and bladder cancer tissue than their corresponding para-carcinoma tissues. This verified that S100A8 could be a predictor of prognosis of breast cancer and bladder cancer patients.

The potential limitations of our meta-analysis include: 1) publication bias because negative results were more difficult to be published and we only searched four online databases and only included English written studies. 2) Heterogeneity of data as shown by our subgroup analysis, where overexpression S100A8 was significantly associated with poor outcome in studies with a sample size greater than 100 but not in studies with a sample size smaller than 100. It is possible that studies with smaller sample size were not statistically stable. 3) Cut-off values were different among these eligible studies, which meant the baseline of overexpression S100A8 may be different. 4) We included two studies that only provided the Kaplan-Meier curve instead of detailed data for HR and 95% CI in survival analysis. Although this increased the amount of data, the accuracy of the meta-analysis may be decreased. 5) The number of included studies was still small. Ideally, a large-size, multicenter study with high-quality will be extremely beneficial for clearly delineating the clinical values of S100A8 in human solid cancers.

In conclusion, our meta-analysis showed that S100A8 expression was a promising predictor and biomarker of prognosis in breast cancer and bladder cancer. Whether or not S100A8 expression could be used as a prognostic biomarker in other cancers requires further study.

## Data Availability Statement

The raw data supporting the conclusions of this article will be made available by the authors, without undue reservation.

## Ethics Statement

The studies involving human participants were reviewed and approved by Medical Ethical Committee of the Zhongnan Hospital of Wuhan University. The patients provided written informed consent to participate in this study.

## Author Contributions

AH conceived and designed the study. AH, WF, and JL performed the literature search and extracted the data. BH, QC, and LC analyzed the data and summarized results. WF, PW, YD, and TM performed the validation. AH drafted the manuscript. YW and MY revised and proofread the manuscript. All authors contributed to the article and approved the submitted version.

## Funding

The study was supported by National Natural Science Foundation of China (No. 81472033 and 30901308), the National Science Foundation of Hubei Province (No. 2013CFB233), Health Commission of Hubei Province scientific research project (No.WJ2019M203), the Application Basic Research Plan Program of Wuhan (No. 2017060201010171), the joint fund project of Hubei Health and Family Planning Commission (No. WJ2018H0028), Hubei Province Health and Family Planning Scientific Research Project (No. WJ2015Q021), the Scientific and Technological Project of Wuhan (No. 2014060101010045), and Training Program of the Science and Technology Innovation from Zhongnan Hospital of Wuhan University (No. cxpy2018031 and No. cxpy20160054), Wuhan University Student Innovation Project (No.MS2017045 and S2018301747).

## Conflict of Interest

The authors declare that the research was conducted in the absence of any commercial or financial relationships that could be construed as a potential conflict of interest.
